# Association of non-high-density lipoprotein cholesterol to high-density lipoprotein cholesterol (NHHR) and sarcopenia in elderly adults

**DOI:** 10.3389/fnut.2025.1614263

**Published:** 2025-06-30

**Authors:** Yi Lin, Xiaocong Shi, Weijia Wu, Xiyi Chen

**Affiliations:** ^1^Department of Endocrinology, The Third Affiliated Hospital of Shanghai University, Wenzhou People’s Hospital, Wenzhou, China; ^2^Department of Cardiovascular and Thoracic Surgery, The Second Affiliated Hospital and Yuying Children’s Hospital of Wenzhou Medical University, Wenzhou, China

**Keywords:** NHHR, lipids, sarcopenia, elderly, NHANES

## Abstract

**Background:**

This study aimed to explore the link between non-high-density lipoprotein cholesterol to high-density lipoprotein cholesterol ratio (NHHR) and sarcopenia in elderly adults using data from the National Health and Nutrition Examination Survey (NHANES).

**Methods:**

The dataset from NHANES 1999–2004. Different statistical analyses, including logistic regression, restricted cubic spline, two-piecewise linear regression, subgroup analyses, and interaction tests models, were used to examine the relationship between NHHR and the risk of sarcopenia.

**Results:**

Total 3,190 elderly participants were included and 676 (21.2%) of them had sarcopenia. In crude model, the odds ratio (OR) of NHHR on sarcopenia was 0.71 [95% confidence interval (CI), 0.66–0.77]. After adjusting for all factors, the ORs for NHHR tertiles 2 and 3 were 0.76 (95% CI: 0.58–0.99) and 0.70 (95% CI: 0.53–0.94), respectively, with a significant trend (*p* = 0.014). An L-shaped relationship between NHHR and sarcopenia was found (P for non-linearity = 0.046), with 3.01 being the inflection point. Subgroup analysis results indicated a stable and persistent association between NHHR and sarcopenia across multiple subgroups, with the exception of diabetes.

**Conclusion:**

We found that NHHR was negatively associated with risk of sarcopenia in elderly adults.

## 1 Introduction

Sarcopenia is a widespread and advancing condition that leads to a rapid decline in skeletal muscle mass and function (1, 2). The pooled prevalence estimates of sarcopenia from 58 distinct study populations across 26 countries varied between 10% and 40%, contingent on the definition used (3). Sarcopenia can lead to a decrease in physical abilities and movement, negatively impact quality of life, and heighten the risk of various negative outcomes such as falls, fractures, and early death (1, 4, 5). Sarcopenia is a significant public health issue linked to substantial healthcare costs, which are anticipated to rise rapidly in aging populations (6). The 2016 assignment of an International Classification of Diseases-10 clinical modification code to sarcopenia highlights the increasing acknowledgment of its clinical significance (7). Therefore, assessing and addressing both traditional and novel risk factors is crucial to mitigate the negative health impacts of sarcopenia.

Fatty acids and lipid metabolism intermediates are crucial in regulating skeletal muscle mass and function (8). Accumulation of these lipids and their derivatives in muscle cells and adjacent areas may lead to lipid toxicity. This accumulation leads to oxidative stress, mitochondrial dysfunction, inflammation, and insulin resistance, adversely affecting muscle health (9). Dyslipidemia could increase the likelihood of sarcopenia through mechanisms such as insulin resistance, inflammation, and oxidative stress. The ratio of non-high-density lipoprotein cholesterol (non-HDL-C) to high-density lipoprotein cholesterol (HDL-C), known as non-high-density lipoprotein cholesterol to high-density lipoprotein cholesterol ratio (NHHR), is a new tool for assessing the lipid spectrum (10). NHHR has demonstrated promising predictive value in assessing the risk of various diseases, including mortality, diabetes, diabetic kidney disease hyperuricemia and carotid atherosclerosis (10–15).

The prevalence of sarcopenia generally rises with age and is particularly elevated among elderly institutionalized populations, suggesting a higher proportion of elderly adults with the condition. This suggested that early identification of sarcopenia in elderly adults was critical to their prognosis. However, the prognostic significance of NHHR for sarcopenia in elderly adults is still uncertain. Therefore, this study aimed to explore the link between NHHR and sarcopenia in elderly adults using data from the National Health and Nutrition Examination Survey (NHANES), with the goal of offering useful insights for disease management.

## 2 Materials and methods

### 2.1 Study population

**FIGURE 1:**
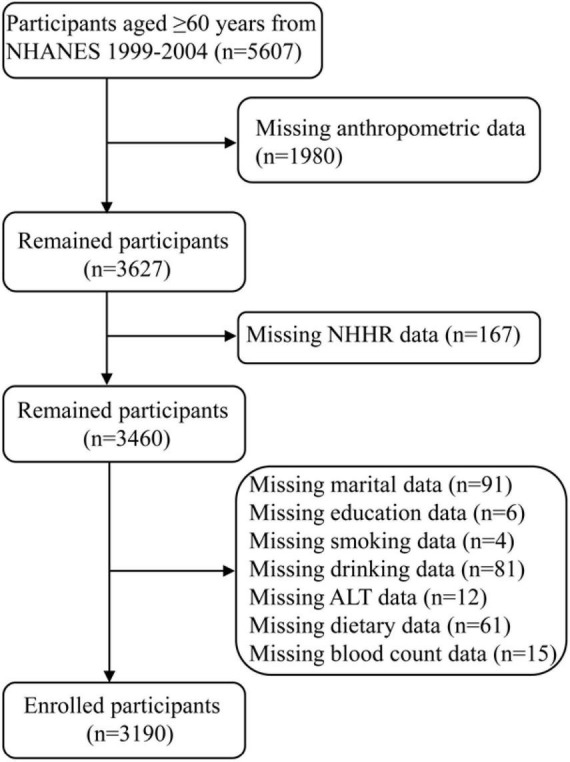
The flowchart of the participant selection.

The NHANES database was a national survey designed to assess the health and dietary habits of non-institutionalized individuals in the United States. The study initially included 5,607 participants aged 60 years and older from the NHANES 1999–2004 dataset. The exclusion criteria included missing data in the following areas: anthropometric measurements, NHHR, and covariates. In the end, total 3,190 elderly participants were included (Figure 1).

### 2.2 Definition of NHHR

Non-high-density lipoprotein cholesterol to high-density lipoprotein cholesterol ratio was determined by the ratio of non-HDL-C (total cholesterol minus HDL-C) to HDL-C (11).

### 2.3 Definition of sarcopenia

Appendicular lean mass (ALM) refers to the total muscle mass in the limbs of both the upper and lower body. The appendicular lean mass index (ALMI) is determined by dividing ALM by the square of the height. For men, sarcopenia is defined as having an ALMI below 7.0 kg/m^2^, while for women, it is below 5.5 kg/m^2^ (16).

### 2.4 Covariates

Demographic parameters included age, sex, race, marital status, and education level. Additionally, we considered various anthropometric, dietary and laboratory covariates, such as smoking status, drinking status, height, weight, body mass index (BMI), waist circumference, waist-to-height ratio (WHtR), ALMI, fat, hypertension, diabetes, protein intake, neutrophil-to-lymphocyte ratio (NLR), aspartate aminotransferase (ALT), alanine aminotransferase (AST), creatinine, uric acid, blood urea nitrogen, total cholesterol and HDL-C. Low waist circumference was defined as <102 cm for men and <88 cm for women. The condition of hypertension was defined by the prescription of antihypertensive drugs, a systolic blood pressure equal to or exceeding 140 mmHg, or a diastolic blood pressure equal to or exceeding 90 mmHg. Diabetes was identified when any of these criteria were satisfied: using diabetes drugs or insulin, or glycated hemoglobin equal to or exceeding 6.5%, or fasting plasma glucose level equal to or exceeding 7.0 mmol/L, or self-reported having diabetes.

### 2.5 Statistical analysis

Continuous variables were represented as either the mean with standard deviation (SD) or the median with interquartile range (IQR), employing the Student’s *t*-test and the Mann-Whitney U test, respectively. Categorical variables were described in terms of patient counts and proportions, with comparisons made using the chi-square test or Fisher’s exact test. The associations between NHHR and sarcopenia were evaluated through three distinct logistic regression models. Package “car” was used to calculate the Variance Inflation Factor (VIF) to estimate the potential multicollinearity. The package of “rcssci” was used to plot the restricted cubic splines for exploration of potential non-linearity. This package can select the optimal knot and identify inflection point automatically. The likelihood ratio test was performed with the “lmtest” package to compare the likelihood ratio of the segmented model with the original linear model. Subgroup analyses were performed to adjust for confounding variables, and interaction tests were applied to assess potential heterogeneity among these subgroups. Sensitivity analysis was performed to test the model stability. Statistical significance was defined as a two-sided *P*-value less than 0.05 (unless otherwise indicated). Bonferroni corrections were used in subgroup interaction tests to addressing potential false positives. Therefore, *P*-values less than adjusted α were defined as statistical significance in subgroup analyses. All analyses were conducted using R software (version 4.4.1).

## 3 Results

Table 1 displayed the characteristics sorted by sarcopenia. Total 3,190 elderly participants were included and 676 (21.2%) of them had sarcopenia. The tertile ranges for NHHR were T1: 0.53–2.50, T2: 2.51–3.58 and T3: 3.59–10.22. Among them, 50.09% were male and 49.91% were female, with a mean age of 70.88 (7.66) years. Participants with sarcopenia were more prone to being older, predominantly White Non-Hispanic, married, smoking, drinking, intaking more protein, having a lower height, weight, BMI, waist circumference, WHtR, fat, NLR, ALT, uric acid, NHHR, having higher HDL-C, and higher prevalence of diabetes. There no difference in sex, education level, hypertension, AST, creatinine, blood urea nitrogen, and total cholesterol.

**TABLE 1:** Baseline characteristics of the study population.

Variables	Total (*n* = 3190)	Non-sarcopenia (*n* = 2514)	Sarcopenia (*n* = 676)	*P*
Age, years	70.88 ± 7.66	70.03 ± 7.34	74.06 ± 7.96	<0.001
Sex				0.536
Male	1598 (50.09)	1267 (50.40)	331 (48.96)	
Female	1592 (49.91)	1247 (49.60)	345 (51.04)	
Race				<0.001
Non-Hispanic White	1852 (58.06)	1385 (55.09)	467 (69.08)	
Non-Hispanic Black	444 (13.92)	420 (16.71)	24 (3.55)	
Mexican-American	692 (21.69)	559 (22.24)	133 (19.67)	
Other	202 (6.33)	150 (5.97)	52 (7.69)	
Marital status				0.006
Married	1973 (61.85)	1587 (63.13)	386 (57.10)	
Other	1217 (38.15)	927 (36.87)	290 (42.90)	
Education level				0.540
Below high school	1306 (40.94)	1038 (41.29)	268 (39.64)	
High school	753 (23.61)	583 (23.19)	170 (25.15)	
Above high school	1131 (35.45)	893 (35.52)	238 (35.21)	
Smoking status				<0.001
Never	1480 (46.39)	1191 (47.37)	289 (42.75)	
Former	1316 (41.25)	1045 (41.57)	271 (40.09)	
Current	394 (12.35)	278 (11.06)	116 (17.16)	
Drinking status				0.019
Never	579 (18.15)	436 (17.34)	143 (21.15)	
Former	953 (29.87)	742 (29.51)	211 (31.21)	
Current	1658 (51.97)	1336 (53.14)	322 (47.63)	
Height, cm	165.32 ± 9.91	165.61 ± 9.98	164.24 ± 9.60	<0.001
Weight, kg	76.55 ± 16.18	80.40 ± 15.30	62.24 ± 10.28	<0.001
BMI, kg/m^2^	27.93 ± 5.00	29.25 ± 4.63	23.00 ± 2.74	<0.001
BMI				<0.001
<25 kg/m^2^	925 (29.00)	401 (15.95)	524 (77.51)	
25–30 kg/m^2^	1330 (41.69)	1179 (46.90)	151 (22.34)	
≥30 kg/m^2^	935 (29.31)	934 (37.15)	1 (0.15)	
Waist circumference, cm	99.53 ± 12.85	102.29 ± 12.10	89.27 ± 10.09	<0.001
Waist circumference				<0.001
Low	1211 (37.96)	729 (29.00)	482 (71.30)	
High	1979 (62.04)	1785 (71.00)	194 (28.70)	
WHtR	0.60 ± 0.08	0.62 ± 0.07	0.54 ± 0.06	<0.001
ALMI, kg/m^2^	7.16 ± 1.36	7.54 ± 1.22	5.76 ± 0.80	<0.001
Fat,%	36.04 ± 7.83	36.51 ± 7.80	34.32 ± 7.70	<0.001
Hypertension				0.093
No	975 (30.56)	750 (29.83)	225 (33.28)	
Yes	2215 (69.44)	1764 (70.17)	451 (66.72)	
Diabetes				<0.001
No	2238 (70.16)	1711 (68.06)	527 (77.96)	
Yes	952 (29.84)	803 (31.94)	149 (22.04)	
Protein intake, g/day	68.14 ± 32.57	69.57 ± 32.79	62.78 ± 31.19	<0.001
NLR	2.37 ± 1.35	2.28 ± 1.24	2.70 ± 1.66	<0.001
ALT, U/L	23.44 ± 37.25	24.17 ± 41.25	20.76 ± 14.49	<0.001
AST, U/L	25.31 ± 30.85	25.39 ± 34.26	25.00 ± 11.19	0.629
Creatinine, umol/L	83.74 ± 43.79	83.39 ± 41.33	85.03 ± 51.95	0.450
Uric acid, umol/L	334.62 ± 86.37	341.24 ± 85.70	310.03 ± 84.42	<0.001
Blood urea nitrogen, umol/L	5.89 ± 2.54	5.88 ± 2.46	5.90 ± 2.79	0.860
Total cholesterol, mmol/L	5.40 ± 1.05	5.41 ± 1.05	5.38 ± 1.07	0.509
HDL-C, mmol/L	1.38 ± 0.42	1.34 ± 0.39	1.53 ± 0.48	<0.001
NHHR	3.21 ± 1.33	3.32 ± 1.34	2.80 ± 1.24	<0.001
NHHR				<0.001
T1	1064 (33.35)	747 (29.71)	317 (46.89)	
T2	1055 (33.07)	853 (33.93)	202 (29.88)	
T3	1071 (33.57)	914 (36.36)	157 (23.22)	

Values are shown as number (%) unless otherwise indicated. BMI, body mass index; ALMI, Appendicular lean mass index; WHtR, waist-to-height ratio; NLR, neutrophil-to-lymphocyte ratio; ALT, Aspartate aminotransferase; AST, Alanine aminotransferase; HDL-C, high-density lipoprotein cholesterol; NHHR, non-HDL-C to HDL-C ratio; NHHR Tertile rang: T1: 0.53, 2.50; T2: 2.51, 3.58; T3: 3.59, 10.22.

Table 2 illustrated the logistic regression analysis of the connection between NHHR and sarcopenia, showing a consistent relationship across different models. All variables of VIF < 5 (Supplementary Table 1), so there was no multicollinearity relationship. In crude model, the odds ratio (OR) of NHHR on sarcopenia was 0.71 [95% confidence interval (CI), 0.66–0.77]. Participants in the lowest NHHR tertile exhibited a higher susceptibility to sarcopenia compared to those in the highest tertile [OR 0.41 (95% CI, 0.33–0.50)]. In model 2, the OR was 0.70 (95% CI, 0.64–0.76) after adjusting for demographic factors (age, sex, race, marital status, education), anthropometric indicators (BMI, waist circumference), lifestyle factors (smoking, drinking), protein intake and clinical variables (hypertension, diabetes, NLR, ALT, AST, creatinine, uric acid, blood urea nitrogen). The ORs for NHHR tertiles 2 and 3 were 0.76 (95% CI: 0.58–0.99) and 0.70 (95% CI: 0.53–0.94), respectively, with a significant trend (*p* = 0.014).

**TABLE 2:** Logistic regression analysis on the association between NHHR and the risk of sarcopenia in elderly adults.

	Crude model		Model 1^a^		Model 2^b^	
	OR (95% CI)	*P*	OR (95% CI)	*P*	OR (95% CI)	*P*
NHHR (continuous)	0.71 (0.66, 0.77)	<0.001	0.70 (0.64, 0.76)	<0.001	0.76 (0.70, 0.83)	<0.001
NHHR (categories)						
T1	Ref.		Ref.		Ref.	
T2	0.56 (0.46, 0.68)	<0.001	0.72 (0.56, 0.93)	0.012	0.76 (0.58, 0.99)	0.042
T3	0.41 (0.33, 0.50)	<0.001	0.65 (0.49, 0.86)	0.002	0.70 (0.53, 0.94)	0.018
P for trend	<0.001		0.001		0.014	

OR, odds ratio; CI, confidence interval.

*^a^*Model 1: adjusted for age, sex, race, marital status, education, BMI, and waist circumference.

*^b^*Model 2: model 1 + smoking status, drinking status, hypertension, diabetes, protein intake, NLR, ALT, AST, creatinine, uric acid, and blood urea nitrogen.

**FIGURE 2:**
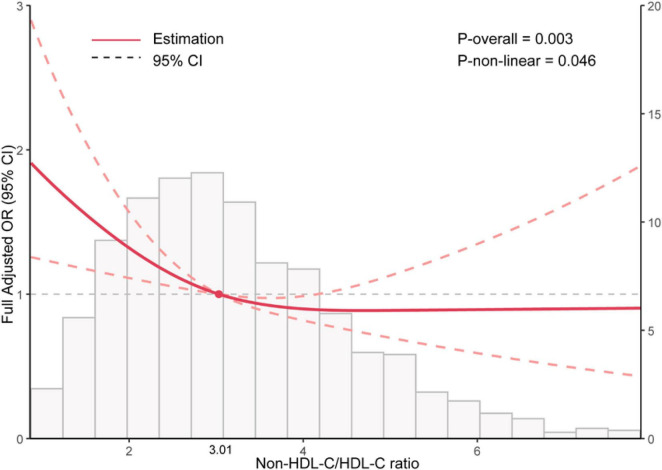
Restricted cubic spline analysis between Non-HDL-C/HDL-C ratio (NHHR) and the risk of sarcopenia in elder adults.

In Figure 2, the restricted cubic spline of OR and 95% CI for the connection between NHHR and sarcopenia was displayed. An L-shaped relationship between NHHR and sarcopenia was found (P for non-linearity = 0.046).

Table 3 indicated an inflection point at 3.01, with 1,600 individuals (50.2%) having a NHHR above this point and 1,590 individuals (49.8%) below it. Each increase in NHHR corresponded to a 29% reduction in the risk of sarcopenia below the inflection point. Nevertheless, the relationship was not statistically significant beyond the inflection point. The likelihood ratio test indicates a statistically significant difference between the segmented model and the original model, with a *P*-value less than 0.05.

**TABLE 3:** Threshold effect analysis of NHHR on the risk of sarcopenia using a two-piecewise linear regression model.

	Adjusted OR (95% CI)	*P*
Fitting by standard linear model	0.76 (0.70, 0.83)	<0.001
Fitting by two-piecewise linear model		
Inflection point	3.01	
<3.01	0.71 (0.54, 0.94)	0.015
≥3.01	0.98 (0.82, 1.17)	0.817
Log-likelihood ratio		<0.001

OR, odds ratio; CI, confidence interval.

Table 4 showed the results of subgroup analysis. Only one person with sarcopenia has a BMI greater than 30. Therefore, merging this group with the 25–30 kg/m^2^ category to form ≥25 kg/m^2^ category. Subgroup analysis results indicated a stable and persistent association between NHHR and sarcopenia across multiple subgroups. Significantly, no major interactions were detected concerning sex, race, marital status, education level, BMI, waist, smoking status, drinking status, and hypertension (all p for interaction > adjusted α). Nevertheless, the strength of the correlation between NHHR and sarcopenia was significantly affected by diabetes (p for interaction = 0.013). The relationship between NHHR and sarcopenia appears to disappear in individuals without diabetes.

**TABLE 4:** Subgroup analyses for the association between NHHR and the risk of sarcopenia in elderly adults.

Variables	OR	95% CI	*P*	*P* for interaction	Adjusted α
Sex				0.522	0.05
Male	0.91	0.80–1.04	0.168		
Female	0.85	0.74–0.99	0.035		
Race				0.918	0.008
Non-Hispanic White	0.9	0.80–1.02	0.096		
Non-Hispanic Black	0.75	0.43–1.25	0.286		
Mexican-American	0.83	0.68–1.01	0.064		
Other	0.88	0.61–1.24	0.472		
Marital status				0.317	0.05
Married	0.89	0.79–1.01	0.07		
Other	0.87	0.75–1.01	0.074		
Education level				0.064	0.017
Below high school	0.95	0.83–1.09	0.462		
High school	0.87	0.71–1.05	0.156		
Above high school	0.79	0.66–0.95	0.016		
BMI				0.514	0.05
<25 kg/m^2^	0.88	0.76–1.02	0.092		
≥25 kg/m^2^	0.85	0.74–0.96	0.01		
Waist				0.639	0.05
Low	0.85	0.75–0.96	0.009		
High	0.88	0.75–1.02	0.104		
Smoking status				0.848	0.017
Never	0.87	0.75–1.01	0.065		
Former	0.87	0.74–1.01	0.072		
Current	0.93	0.74–1.16	0.508		
Drinking status				0.708	0.017
Never	0.95	0.76–1.18	0.662		
Former	0.92	0.77–1.09	0.334		
Current	0.83	0.72–0.95	0.009		
Hypertension				0.198	0.05
No	0.81	0.68–0.95	0.012		
Yes	0.92	0.82–1.03	0.165		
Diabetes				0.013	0.05
No	0.81	0.72–0.91	<0.001		
Yes	1.05	0.87–1.26	0.596		

Adjusted for age, sex, race, marital status, education, BMI, waist circumference, smoking status, drinking status, hypertension, diabetes, protein intake, NLR, ALT, AST, creatinine, uric acid, and blood urea nitrogen. Stratified variables themselves were not adjusted in the subgroup analysis.

Table 5 showed the sensitivity analysis on the association between NHHR and sarcopenia. Participants with missing covariate data were included. Total 3,460 participants were included in the sensitivity analysis. In crude model, the OR of NHHR on sarcopenia was 0.71 [95% CI, 0.66–0.76]. Participants in the lowest NHHR tertile exhibited a higher susceptibility to sarcopenia compared to those in the highest tertile [OR 0.40 (95% CI, 0.33–0.49)]. In model 2, the OR was 0.77 (95% CI, 0.70–0.93) after adjusting for all factors. The ORs for NHHR tertiles 2 and 3 were 0.76 (95% CI: 0.58–0.99) and 0.70 (95% CI: 0.52–0.94), respectively, with a significant trend (*p* = 0.013).

**TABLE 5:** Sensitivity analysis on the association between NHHR and the risk of sarcopenia in elderly adults.

	Crude model		Model 1^a^		Model 2^b^	
	OR (95% CI)	*P*	OR (95% CI)	*P*	OR (95% CI)	*P*
NHHR (continuous)	0.71 (0.66, 0.76)	<0.001	0.70 (0.65, 0.75)	<0.001	0.77 (0.70, 0.83)	<0.001
NHHR (categories)						
T1	Ref.		Ref.		Ref.	
T2	0.58 (0.48, 0.70)	<0.001	0.72 (0.56, 0.92)	0.010	0.76 (0.58, 0.99)	0.041
T3	0.40 (0.33, 0.49)	<0.001	0.64 (0.49, 0.84)	0.001	0.70 (0.52, 0.94)	0.017
*P* for trend	<0.001		0.001		0.013	

OR, odds ratio; CI, confidence interval.

*^a^*Model 1: adjusted for age, sex, race, marital status, education, BMI, and waist circumference.

*^b^*Model 2: model 1 + smoking status, drinking status, hypertension, diabetes, protein intake, NLR, ALT, AST, creatinine, uric acid, and blood urea nitrogen.

## 4 Discussion

In this study, we investigated the connections of NHHR and sarcopenia in elderly people. We found that NHHR was negatively associated with sarcopenia. The restricted cubic spline analysis revealed L-shaped relationship between NHHR and sarcopenia in elderly people, identifying NHHR = 3.01 as the optimal threshold. The subgroup analysis results showed consistent links between NHHR and sarcopenia in different subgroups. The sensitivity analyses verified the robustness of the results.

Non-high-density lipoprotein cholesterol to high-density lipoprotein cholesterol ratio is a new indicator for evaluating lipid abnormalities in atherosclerosis, integrating information on both harmful and protective lipid particles. It potentially reflects the balance between these lipoprotein classes and is often elevated in obese populations (10). In this study, we found that increased NHHR levels were associated with lower risk of sarcopenia among elderly adults. However, the direction of associations in this study is inconsistent with current evidence. Prior studies have highlighted that NHHR is linked to a heightened risk of sarcopenia in both young adults (17) and cancer patients (18). The potential reasons for discrepancies maybe the diagnostic criteria (use of ALM/BMI vs. ALM/height^2^). According to the European Working Group on Sarcopenia in Older People-2 (EWGSOP2), as mentioned earlier, muscle mass is linked to body size, allowing for various adjustments based on body size (19). However, no recommendation is made to account for body size, though adjustments are possible if relevant normative population data is available. Whether based on EWGSOP (20), EWGSOP2 (16), the Asian Working Group for Sarcopenia (AWGS) (21), the International Working Group on Sarcopenia (IWGS) (22), or the Foundations for the National Institutes of Health (FNIH) (23), it is recommended that ALM or ALM/height^2^ be used instead of ALM/BMI. Because they used ALM/BMI to define sarcopenia, it is absurd to observe in the baseline table that the incidence of sarcopenia is increased with increasing BMI. A meta-analysis indicated an inverse relationship between BMI and sarcopenia risk, while muscle mass showed a positive correlation with BMI (24). When muscle quantity was adjusted, a higher BMI was related to a heightened risk of sarcopenia (25). In the above 2 articles, sarcopenia was not adjusted for muscle quantity, and it was shown that the incidence of sarcopenia increased with increasing BMI, which is not reasonable. Recent China Health and Retirement Longitudinal Study (CHARLS) studies have shown that elevated HDL-C have significant clinical value in the risk assessment of sarcopenia (26, 27). In the formula of NHHR, it can be found that HDL-C is negatively correlated with NHHR. This can also verify the rationality of the negative correlation between NHHR and sarcopenia from this side. It is also possible that the different study populations may have contributed to the different results. Multicenter studies with larger sample sizes are needed to validate the relationship between NHHR and sarcopenia.

Previous studies have shown that NHHR is a “bad indicator” associated with increased risks of chronic kidney disease (28), infertility (29), cardiometabolic disease (30), non-alcoholic fatty liver disease (31), depressive symptoms (32), and gallstones, in contrast to traditional cholesterol indices (33). However, recent cohort studies indicate that elevated NHHR levels reduce cardiovascular risk and all-cause mortality in cancer survivors (34). A study by Yu et al. demonstrated a U-shaped relationship between NHHR and all-cause mortality, and an L-shaped relationship with cardiovascular mortality in individuals with diabetes or prediabetes (11). These studies indicate that NHHR levels should be kept within an appropriate range. Extreme NHHR levels are linked to higher mortality risk. Interestingly, although the diseases were different, our results were consistent with above evidence, implying NHHR was a “bad indicator” that needs to be further considered.

To discover the essential reality, we carried out a subgroup analysis in this research to optimize data usage. In subgroup analyses, we found that there was an effect of diabetes on the association between NHHR and sarcopenia. Within the diabetes subgroup, the correlation was more pronounced among non-diabetic individuals, whereas it was attenuated and not statistically significant among diabetic individuals. This observation may be attributable to irreversible muscle damage resulting from prolonged metabolic disturbances in individuals with diabetes. Furthermore, the intricate metabolic compensation mechanisms or other associated complications in diabetic patients may further obscure the direct relationship between NHHR and sarcopenia (35). These findings indicate that NHHR may exhibit greater sensitivity in non-diabetic populations, thereby providing a more precise reflection of the potential impact of lipid metabolism disorders on muscle strength.

Non-high-density lipoprotein cholesterol to high-density lipoprotein cholesterol ratio frequently shows elevated levels in obese individuals, indicating lipid abnormalities. This correlation is due to obesity being linked to increased levels of low-density lipoprotein cholesterol (LDL-C) and very low-density lipoprotein cholesterol (VLDL-C), alongside a decrease in HDL-C levels, which results in a rise in NHHR (36). Obesity frequently disrupts lipid metabolism, altering fat distribution and utilization in the body. These alterations elevate LDL-C and VLDL-C levels while reducing HDL-C levels, thereby further raising NHHR (37). A hypothesis of underlying pathophysiologic mechanism suggested that dysfunctional HDL-C increases levels of proinflammatory, which induces cellular degradation through the mechanism of skeletal muscle mitochondrial dysfunction. This results in elevated reactive oxygen species, triggering the ubiquitin proteasome cascade and enhancing muscle proteolysis, ultimately reducing muscle mass (38–42). The mechanisms linking NHHR to sarcopenia risk require further investigation.

This research identifies NHHR as a potential new biomarker that could help clinicians evaluate sarcopenia, opening up opportunities for future research and therapy development. The introduction of new lipid metrics enhances health education for sarcopenic patients and aids in creating personalized treatment plans. Nonetheless, the study has certain limitations. Firstly, while researchers identified a potential link between NHHR levels and sarcopenia, the cross-sectional approach prevents the definitive formation of a causal connection. Secondly, while low muscle mass is a key aspect of sarcopenia, the current database’s absence of data on muscle strength and physical performance does not completely clarify the link between NHHR and sarcopenia. Although the stratified analysis addressed certain confounding factors, it did not account for all potential confounders. The study’s reliance on United States-based data necessitates further research to determine the findings’ applicability to other regions.

## 5 Conclusion

The results of this study indicate that the NHHR is a significant predictive marker for assessing the risk of sarcopenia among older adults. Within a nationally representative cohort of elderly individuals in the United States, an L-shaped relationship was identified between NHHR and the risk of sarcopenia. Regular monitoring of NHHR could enhance the assessment of sarcopenia risk and prognosis in this population.

## Data Availability

Publicly available datasets were analyzed in this study. This data can be found here: www.cdc.gov/nchs/nhanes/.
