# Supplementary material for: Association of non-high-density lipoprotein cholesterol to high-density lipoprotein cholesterol (NHHR) and sarcopenia in elderly adults
Source: Front Nutr. 2025 Jun 30;12:1614263. doi: 10.3389/fnut.2025.1614263 (PMC12256216; doi:10.3389/fnut.2025.1614263)
Supplement: Supplementary file 1 [file Table_1.docx]

Supplementary Table 1. The variance inflation factor (VIF).

| Variables | VIF |
| --- | --- |
| NHHR | 1.203 |
| Age | 1.459 |
| Sex | 1.923 |
| Race | 1.648 |
| Marital status | 1.238 |
| Education level | 1.407 |
| Smoking status | 1.422 |
| Drinking status | 1.351 |
| BMI | 3.359 |
| Waist circumference | 3.866 |
| Hypertension | 1.138 |
| Diabetes | 1.110 |
| Protein intake | 1.160 |
| NLR | 1.114 |
| ALT | 4.128 |
| AST | 4.125 |
| Creatinine | 1.424 |
| Uric acid | 1.361 |
| Blood urea nitrogen | 1.581 |
